# The efficacy of adebrelimab compared with durvalumab and atezolizumab in untreated extensive-stage small-cell lung cancer: a survival analysis of reconstructed patient-level data

**DOI:** 10.3389/fimmu.2023.1185577

**Published:** 2023-05-05

**Authors:** Bi-Cheng Wang, Chen Fu, Guo-He Lin

**Affiliations:** ^1^ Cancer Center, Union Hospital, Tongji Medical College, Huazhong University of Science and Technology, Wuhan, China; ^2^ Department of Dermatology, Wuhan No.1 Hospital, Wuhan, China; ^3^ Department of Oncology, the Second Affiliated Hospital of Anhui Medical University, Hefei, China

**Keywords:** PD-L1 inhibitor, survival, first-line, extensive-stage, small-cell lung cancer

## Abstract

**Background:**

Adebrelimab showed excellent efficacy in the first-line treatment for extensive-stage small-cell lung cancer (ES-SCLC). However, whether adebrelimab is superior to durvalumab and atezolizumab remains unclear. Therefore, we, in this study, aimed to compare the survival data of adebrelimab (CAPSTONE-1 trial) with durvalumab (CASPIAN trial) and atezolizumab (IMpower133 trial) in the first-line setting of ES-SCLC patients.

**Methods:**

Online databases, including PubMed, Embase, Web of Science, and Cochrane CENTRAL, were systematically searched on December 2, 2022. The *metaSurvival* and *IPDfromKM* methods were used to analyze the summary survival curves and the reconstructed patient-level data. The main endpoints were median overall survival (OS) and progression-free survival (PFS).

**Results:**

In this analysis, survival data in the CASPIAN, IMpower133, and CAPSTONE-1 trials were collected from five published studies. The pooled median OS and PFS were 14.0 months (95% CI 11.2-16.6) and 5.6 months (95% CI 4.7-6.7) when ES-SCLC patients received chemotherapy (etoposide and cisplatin/carboplatin) and anti-PD-L1 therapy. Based on the reconstructed patient-level data, adebrelimab significantly prolonged survival outcomes against atezolizumab (OS: Hazard ratio [HR]0.76, 95% CI 0.60-0.95; PFS: HR 0.67, 95% CI 0.54-0.83) and durvalumab (OS: HR 0.75, 95% CI 0.60-0.92).

**Conclusion:**

For previously untreated ES-SCLC patients, longer survival time might be benefited from adding adebrelimab to etoposide-platinum chemotherapy. In future studies, further real-world evidence or head-to-head clinical trials are warranted to confirm the differences between the PD-L1 inhibitors.

## Introduction

The prognosis of extensive-stage small-cell lung cancer (ES-SCLC) remains poor (1). In the era of chemotherapy, etoposide plus platinum was the standard of care. However, the median overall survival (OS) of ES-SCLC might be hard to exceed one year. Additionally, patients, who received first-line chemotherapy, suffered disease progression within approximately six months.

With the exploration and development of immunotherapy, over one year of OS in ES-SCLC patients is coming true (2). Adding anti-PD-L1 therapy to chemotherapy has revolutionized the first-line treatment framework for ES-SCLC. In the CASPIAN trial, the addition of durvalumab to chemotherapy significantly improved the OS and progression-free survival (PFS) compared with placebo plus chemotherapy (3, 4). Subsequently, the IMpower133 trial demonstrated the clinical benefits of atezolizumab in patients with ES-SCLC (5, 6). Based on these two trials, anti-PD-L1 therapy plus etoposide-platinum chemotherapy has become the optimal first-line treatment option for ES-SCLC (2).

The pace of progress continues. ES-SCLC patients in the CAPSTONE-1 trial treated with adebrelimab and chemotherapy achieved a 15.3 months OS with an acceptable safety profile. Nevertheless, the median PFS in all these trials remained within six months (7). Based on these trials, physicians and patients could be confused about which PD-L1 inhibitor might be more suitable for ES-SCLC patients (3, 5, 7).

At the current stage, physicians and patients are eager to ask about the overall benefits in the era of anti-PD-L1 plus chemotherapy and the optimal option of PD-L1 inhibitors because no direct head-to-head trials have been performed across these treatments (adebrelimab vs. durvalumab vs. atezolizumab). In this study, we reconstructed the patient-level survival data in the CASPIAN, IMpower133, and CAPSTONE-1 trials using a new indirect comparison method, the *IPDfromKM* method (8, 9). Through this survival analysis, we intended to provide valuable survival information and inform readers more about this new area of original research.

## Methods

### Literature search

A systematic literature search was carried out to identify the randomized controlled trials eligible for the survival analysis. This search was performed in PubMed, Web of Science, Embase, and Cochrane CENTRAL on December 2, 2022. Search terms included “small-cell lung cancer OR SCLC”, “extensive-stage OR extensive-disease”, “immunotherapy OR PD-L1” and “first-line OR previously untreated OR treatment-naïve”. Relevant records in the bibliography were searched for more eligible trials.

### Inclusion and exclusion criteria

This analysis was conducted according to the Preferred Reporting Items for Systematic Reviews and Meta-analyses (PRISMA) guideline (10). The inclusion criteria were: (1) treatment-naïve ES-SCLC patients; (2) patients in the treatment arm received anti-PD-L1 therapy plus etoposide-platinum chemotherapy; (3) placebo-controlled randomized clinical trial; (4) primary or update time-to-event data reported as a Kaplan-Meier curve. Meeting abstracts and *post-hoc* subgroup analyses were excluded due to immature data and unbalanced backgrounds.

### Data extraction

Detailed therapeutic regimens, doses, and cycles were collected. Kaplan-Meier curves were captured from enrolled trials. Scanlt software (version 2.0.8.0) was used for digitizing the Kaplan-Meier curves.

### Statistical analysis

The *metaSurvival* package of R software (version 4.2.2) was applied to generate the synthesized OS and PFS curves, indicating the overall efficacy of chemotherapy plus anti-PD-L1 therapy in patients with ES-SCLC (11). With the comparisons between PD-L1 inhibitors, *IPDfromKM* method was used to reconstruct the individual patient data (8). Hazard ratio (HR) and 95% CI were carried out by Cox statistics.

## Results

**Figure 1:**
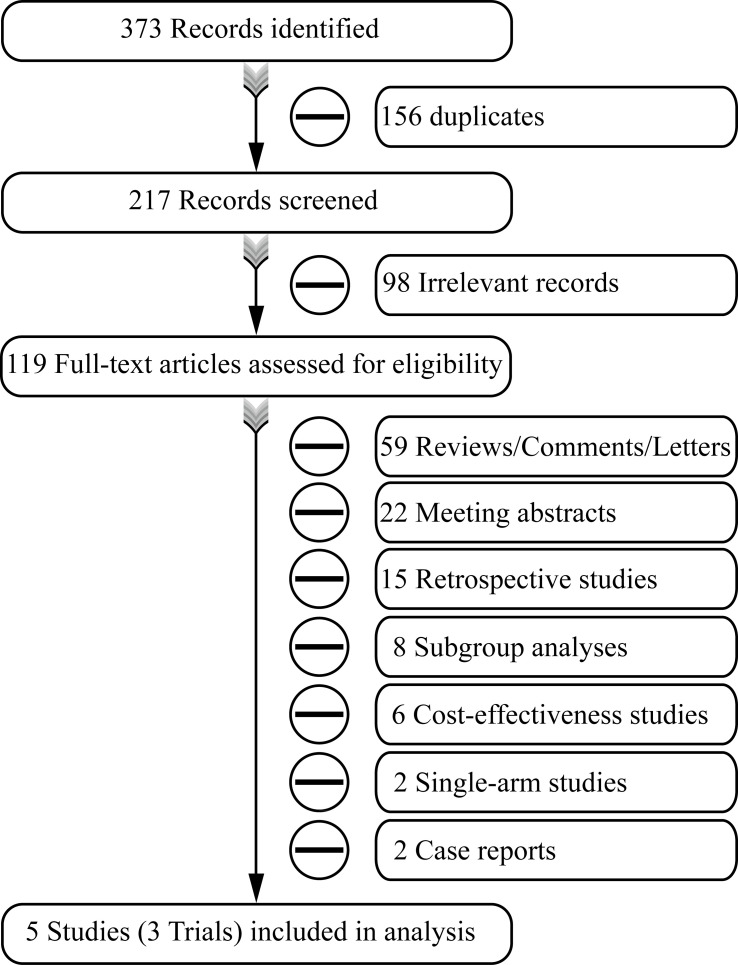
Flowchart of the selection process.

Through searching the online databases, we identified 373 records. After the systematic screening, three placebo-controlled randomized clinical trials reported in five studies (CASPIAN, IMpower133, and CAPSTON-1) were eligible for further survival analysis (**Figure 1**) (3–7).

**Figure 2:**
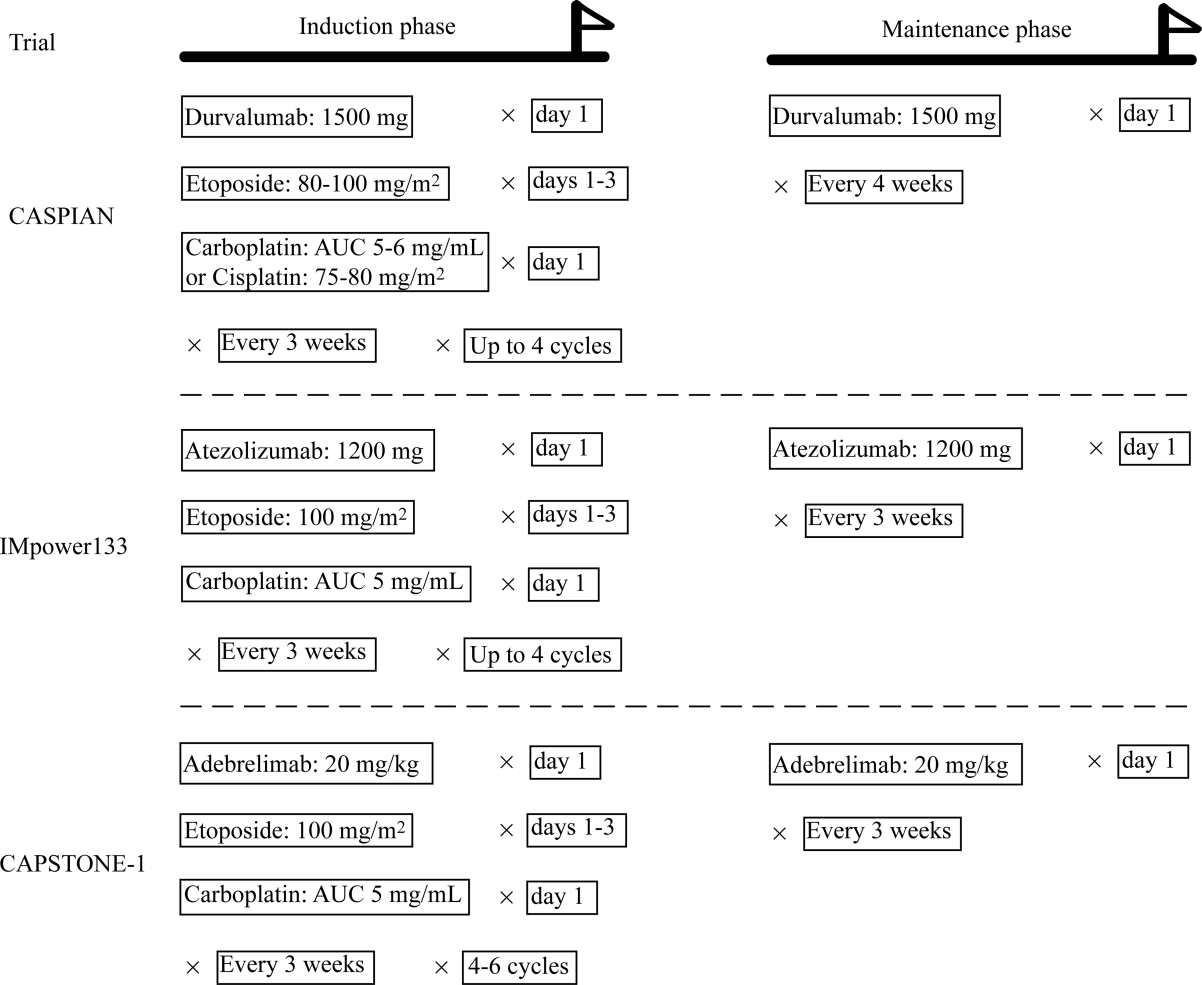
Therapeutic strategies in the enrolled trials.

For enrolled ES-SCLC patients, treatment included induction and maintenance phases (**Figure 2**). During the induction phase, patients in the CASPIAN and IMpower133 trials were treated with up to four cycles of durvalumab/atezolizumab and etoposide-platinum chemotherapy. In the CAPSTONE-1 trial, the cycles ranged from four to six. Another difference existed in the maintenance phase. Atezolizumab and adebrelimab were administrated every three weeks, while durvalumab was prescribed every four weeks.

**Figure 3:**
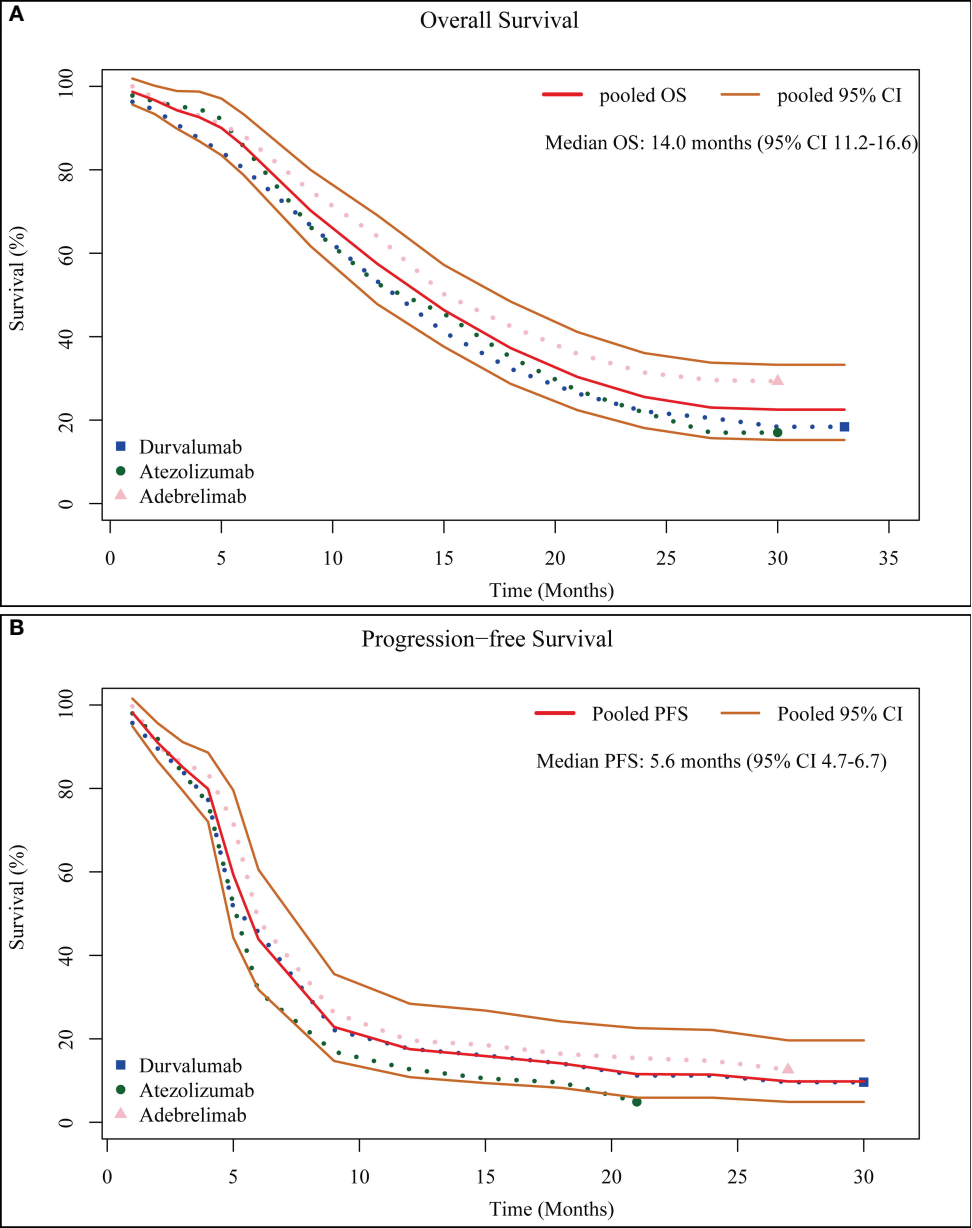
Pooled Kaplan-Meier OS **(A)** and PFS **(B)** curves.

Although the ES-SCLC patients were treated with chemotherapy and anti-PD-L1 therapy, the survival outcomes differed between the trials. The median OS was 12.9 months (95% CI 11.3-14.7) in the durvalumab group, 12.3 months (95% CI 10.8-15.8) in the atezolizumab group, and 15.3 months (95% CI 13.2-17.5) in the adebrelimab group; The median PFS was 5.1 months (95% CI 4.7-6.2) in the durvalumab group, 5.2 months (95% CI 4.4-5.6) in the atezolizumab group, and 5.8 months (95% CI 5.6-6.9) in the adebrelimab group. Subsequently, we synthesized the survival data from these three trials to better understand the overall efficacy of combining chemotherapy with immunotherapy in the first-line treatment of ES-SCLC patients. The pooled median OS and PFS were, respectively, 14.0 months (95% CI 11.2-16.6) and 5.6 months (95% CI 4.7-6.7) (**Figure 3**).

**Figure 4:**
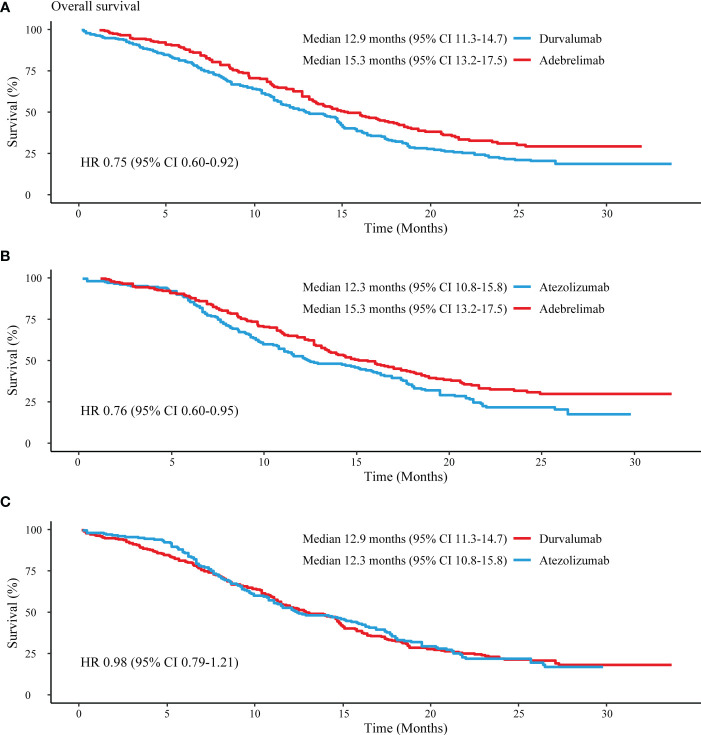
Reconstructed median OS comparisons between PD-L1 inhibitors. **(A)** Adebrelimab vs. Durvalumab; **(B)** Adebrelimab vs. Atezolizumab; **(C)** Atezolizumab vs. Durvaluamb.

Comparisons between PD-L1 inhibitors were conducted based on reconstructed patient-level data. In terms of OS, adebrelimab significantly decreased the risk of death versus durvalumab (HR 0.75, 95% CI 0.60-0.92) and atezolizumab (HR 0.76, 95% CI 0.60-0.95) (**Figures 4A, B** **)**. Durvalumab and atezolizumab showed a comparable OS in treating ES-SCLC patients (HR 0.98, 95% CI 0.79-1.21) (**Figure 4C**). Adebrelimab had higher 1-year and 2-year OS rates (64%, 95% CI 58-70; 31%, 95% CI 25-39) compared with durvalumab (53%, 95% CI 47-59; 22%, 95% CI 17-28) and atezolizumab (53%, 95% CI 46-60; 22%, 95% CI 16-29).

**Figure 5:**
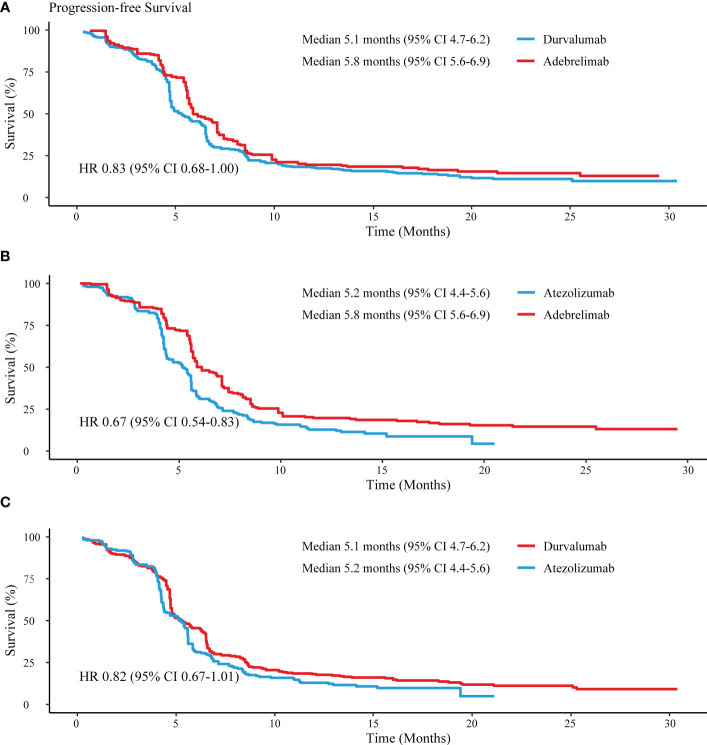
Reconstructed median PFS comparisons between PD-L1 inhibitors. **(A)** Adebrelimab vs. Durvalumab; **(B)** Adebrelimab vs. Atezolizumab; **(C)** Atezolizumab vs. Durvaluamb.

Regarding the reconstructed PFS, adebrelimab (HR 0.83, 95% CI 0.68-1.00) and atezolizumab (HR 0.82, 95% CI 0.67-1.01) showed similar risks of disease progression or death against durvalumab (**Figures 5A, C** **)**. Nevertheless, adebrelimab achieved a lower risk of disease progression or death than atezolizumab (HR 0.67, 95% CI 0.54-0.83) (**Figure 5B**). Additionally, patients in the adebrelimab group showed the highest PFS rates at 6 months (50%, 95% CI 44-57) and 12 months (20%, 95% CI 15-26), followed by durvalumab (46%, 95% CI 40-52; 18%, 95% 14-23) and atezolizumab (32%, 95% CI 26-39; 13%, 95% CI 9-19).

## Discussion

In this original patient-level survival analysis, we synthesized the OS and PFS in ES-SCLC patients treated with anti-PD-L1 therapy plus chemotherapy, with a median OS of 14.0 months (95% CI 11.2-16.6) and a median PFS of 5.6 months (95% CI 4.7-6.7). In addition, patients in the adebrelimab group showed the lowest risks of death or disease progression and highest survival rates compared with durvalumab and atezolizumab. However, it appears to be a debate on whether adebrelimab is superior to durvalumab and atezolizumab.

For patients in the placebo group of the CAPSTONE-1 trial, the median OS was 12.8 months, much longer than the reported data in the CASPIAN (10.5 months) and IMpower133 (10.3 months) trials. Except for the comparable rates of liver metastasis, brain metastasis, and stage IV disease in these three trials, the most apparent difference was that patients in the CAPSTONE-1 received antiangiogenic drugs (24% in the adebrelimab groups and 30% in the placebo group). Nevertheless, the authors did not make any more explanations or discussions on the administration of antiangiogenic therapy. The prolonged OS in the CAPSTONE-1 trial might be partly attributed to antiangiogenic agents (12–14).

Considering OS data could be impacted by subsequent second or later-line treatments, we suggest that median PFS and PFS rate might be more suitable to be applied to assess the efficacy of PD-L1 as the first-line treatment for ES-SCLC (15). Another reason was that PFS data could directly reflect the improvement achieved by adding anti-PD-L1 therapy to chemotherapy. In the CASPIAN trial, the median PFS was 5.1 months in the durvalumab group versus 5.4 months in the placebo group, and the rates of PFS at 12 months were 17.9% in the durvalumab group and 5.3% in the placebo group; In the IMpower133 trial, the median PFS was 5.2 months in the atezolizumab group versus 4.3 months in the placebo group, and the rates of PFS at 12 months were 12.6% in the atezolizumab group and 5.4% in the placebo group; In the CAPSTONE-1 trial, the median PFS was 5.8 months in the adebrelimab group versus 5.6 months in the placebo group, and the rates of PFS at 12 months were 19.7% in the adebrelimab group and 5.9% in the placebo group. According to these results, the atezolizumab group achieved a wider gap (0.9 months) than the durvalumab (-0.3 months) and adebrelimab (0.2 months) groups. Although median PFS failed to be vastly elevated (less than one month), the PFS rates at 12 months were improved. Therefore, PFS data can be considered as primary endpoints in studies investigating the first-line treatment of ES-SCLC.

Regarding the subgroup analyses, more details deserve our attention. Less than 65 years old patients might benefit from adebrelimab, while patients ≥ 65 could choose durvalumab and atezolizumab. In addition, patients with brain or liver metastasis might be hard to profit from adding anti-PD-L1 therapy to chemotherapy owing to the short lifetime. However, we should read the results of subgroup analyses rigorously because the well-balanced backgrounds of patients were lacking.

Physicians and patients may not be satisfied with the current anti-PD-L1 therapy and chemotherapy. There is a long way to prolong the PFS and OS. During the maintenance phase, the addition of radiotherapy to anti-PD-L1 therapy could effectively treat the primary lung or distant metastatic lesions (16, 17). Moreover, antiangiogenic agents have been reported to enhance the effects of immunotherapy in ES-SCLC patients (12, 18).

This survival analysis had both advantages and limitations. Data in the placebo groups were not taken into the analysis, which might increase the reliability of our findings. A Bayesian or frequency network analysis could solve the problem, but it is not feasible at the current stage due to the limited enrolled trials. However, readers can intuitively understand the survival differences between the PD-L1 inhibitors from our reconstructed survival curves. Combining *IPDfromKM* method with Bayesian network analysis is warranted to balance the inhomogeneity among trials. Additionally, we did not intend to further analyze the immune-related adverse events, mainly because PD-L1-related toxicities could be well controlled.

Finally, anti-PD-L1 therapy has improved the survival time of ES-SCLC patients. Although our results showed that patients in the adebrelimab group achieved the most prolonged survival benefits, durvalumab and atezolizumab have their strong points. We hope future studies can provide more real-world comparisons and effective therapeutic strategies.

## Data availability statement

The original contributions presented in the study are included in the article/supplementary material. Further inquiries can be directed to the corresponding author.

## Author contributions

Study design: B-CW. data extraction: B-CW and CF. data analysis: B-CW and CF. Manuscript writing and edition: all authors. All authors contributed to the article and approved the submitted version.
